# Transcriptomic Responses to Koi Herpesvirus in Isolated Blood Leukocytes from Infected Common Carp

**DOI:** 10.3390/v16030380

**Published:** 2024-02-28

**Authors:** Irene Cano, Ellen Blaker, David Hartnell, Audrey Farbos, Karen A. Moore, Adele Cobb, Eduarda M. Santos, Ronny van Aerle

**Affiliations:** 1 International Centre of Excellence for Aquatic Animal Health, Cefas Laboratory, Dorset DT4 8UB, UK; ellen.blaker@cefas.gov.uk (E.B.); david.hartnell@cefas.gov.uk (D.H.); adele.cobb@cefas.gov.uk (A.C.); ronny.vanaerle@cefas.gov.uk (R.v.A.); 2 Centre for Sustainable Aquaculture Futures, University of Exeter, Exeter EX2 4TH, UK; e.santos@exeter.ac.uk; 3 Biosciences, Faculty of Life and Health Sciences, University of Exeter, Exeter EX2 4TH, UK; a.farbos@exeter.ac.uk (A.F.); k.a.moore@exeter.ac.uk (K.A.M.)

**Keywords:** koi herpesvirus (KHV, CyHV-3), common carp, transcriptomics, blood leukocyte, M2 macrophage, toll-like receptors, immunosuppression, pattern recognition receptors, inflammation

## Abstract

Koi herpesvirus (KHV, CyHV-3) causes severe economic losses in carp farms. Its eradication is challenging due to the establishment of latency in blood leukocytes and other tissues. To understand the molecular mechanisms leading to KHV infection in leukocytes, common carp were bath-exposed to KHV at 17 °C. After confirming the presence of viral transcripts in blood leukocytes at ten days post infection, RNA-Seq was performed on peripheral blood leukocytes on the Illumina NovaSeq. KHV infection triggered a robust immune response mediated by pattern recognition receptors, mainly toll-like receptors (*tlr2*, *tlr5*, tlr7, and *tlr13*), *urokinase plasminogen activator surface receptor*-like, galectin proteins, and lipid mediators such as *leukotriene B4 receptor 1*. Enriched pathways showed increased mitochondria oxidative phosphorylation and the activation of signalling pathways such as mitogen-activated protein kinases (MAPKs) and vascular endothelial growth factor (VEGF). KHV-infected leukocytes showed low production of reactive oxygen species (ROS) and glutathione metabolism, high iron export and phagocytosis activity, and low autophagy. Macrophage polarization was deduced from the up-regulation of genes such as *arginase non-hepatic 1*-like, *macrophage mannose receptor-1*, *crem*, *il-10*, and *il-13* receptors, while markers for cytotoxic T cells were observed to be down-regulated. Further work is required to characterise these leukocyte subsets and the molecular events leading to KHV latency in blood leukocytes.

## 1. Introduction

Koi herpesvirus (KHV), also known as cyprinid herpesvirus 3 (CyHV-3) or carp interstitial nephritis gill necrosis virus, is the causative agent of koi herpesvirus disease (KHVD), a severe disease responsible for causing high mortality rates in ornamental koi and common carp (*Cyprinus carpio* L.). The disease was first reported in Israel in 1997 and was soon observed worldwide, except in Australia, due to the intense global trade of koi and common carp [1]. In 2006, KHVD was made notifiable by the World Organisation for Animal Health (WOAH).

KHV primarily infects epithelial cells of skin and gills, after which it becomes viraemic, causing systemic infection and finally infecting cells of the central nervous system [2]. The onset and severity of the disease are highly influenced by water temperature, with an optimal water temperature between 16 and 25 °C. Infections at lower temperatures often result in asymptomatic infections and reduced pathology [3].

KHV belongs to the family Alloherpesviridae within the order Herpesvirales. Two main lineages, European and Asian, have been identified based on a variable number of tandem repeats (VNTR) analysis [4]. With a large, enveloped, double-stranded linear DNA molecule of 298 kbp in length [5], the KHV genome encodes for at least 156 predicted genes in open reading frames (ORFs) [6]. The virion is formed by forty structural proteins, including the immunogenic type 3 membrane protein expressed by ORF81 [6]. Non-structural viral proteins such as interleukin-10 (IL10, ORF134), lipoprotein (ORF68), tumour necrosis factor receptor (TNFR-1, ORF4L) and TNF-2 (ORF12) have been suggested to play a role in the virus survival within the host [7]. However, very little is known about the interaction of these viral proteins with the host immune system.

During the productive phase of the infection, KHV triggers the activation of signalling pathways mainly mediated by interleukin family members and interferon-gamma (IFN)-γ2 [8]. Despite initiating inflammatory responses, KHV subversion of the host’s complement system in mucosal sites allows for long periods of viral shedding [9] and facilitates secondary infections by compromising the mucosal barrier of the skin and the epithelium tight junctions by down-regulating mucin, defensin, and claudin proteins [10]. Breeding selection for KHV resistance has resulted in differences in the immune response between resistant and susceptible families. In particular, resistance to KHV has been linked to autophagy, phagocytosis, cytotoxicity, virus blockage by lectins and mucin, higher expression of immune-related genes, complement activation, MHC class I-restricted antigen presentation, and the development of adaptive mucosal immunity [11,12,13]. Interestingly, differences in the leukocyte population have also been observed among resistant and susceptible carp families, with a higher presence of cxcl8/il8 chemokines and neutrophils in resistant carp and a higher presence of T-cells and macrophages in susceptible families [14]. KHV infection elicits a specific humoral response characterised by high-affinity B-cells [15].

Like many herpesviruses, KHV can lead to life-long infection through latency [7]. Peripheral blood leukocytes, such as IgM-positive B lymphocytes, and other tissues, such as neurons, have been identified as cell targets in which KHV becomes latent [16,17]. In carp, latent KHV can be reactivated and enter productive replication after the fish experience stress, such as when exposed to increased water temperature [16]. Thus, despite the key role of blood leukocytes in initiating inflammatory responses and maintaining KHV infection through latency, the molecular mechanisms leading to KHV infection in leukocytes remain poorly understood. This study aimed to identify critical molecular processes occurring during KHV infection in blood leukocytes before a humoral response occurs. We hypothesise that KHV infection may promote leukocyte subset differentiation involved in the inflammatory response and establishment of latency. To address this hypothesis, we infected common carp with KHV and used RNA-Seq to document the molecular responses to infection in isolated blood leucocytes; the information delivered from this study enhances our understanding of herpesvirus infection in aquatic species and provides valuable evidence to develop therapeutic and vaccinology strategies.

## 2. Materials and Methods

### 2.1. KHV Inoculum Preparation

A European KHV isolate, K250, was obtained from a crucian carp KHVD outbreak in England in 2007. KHV was isolated in KF1 cells (ECACC 10072801, Merck, Gillingham, UK), and the presence of the virus was confirmed via Sanger sequencing of a specific PCR product targeting the viral thymidine kinase gene [18]. K250 was propagated on a common carp brain (CCB) cell line (ECACC 10072802) at 20 °C in EMEM media supplemented with two mM glutamine, 1% non-essential amino acids, 2% foetal bovine serum (FBS), and 10 mM HEPES (Merck, Gillingham, UK). Viral particles were harvested from the supernatant of cells showing cytopathic effects via centrifugation at 4000× *g* for 15 min in a Falcon 6/300 centrifuge ( MSE, Cholet, France) and the viral titre stock inferred via the 50% tissue culture infective dose (TCID50) [19].

### 2.2. Carp Experimental Infection and Blood Samples

Twenty-four specific pathogen-free (SPF) immature mixed-sex common carp, weighing ~400 g, were split into four tanks of six fish each and acclimated to 17 °C. Two tanks were exposed to 15 mL of KHV isolate K250 at ~10^8^ TCID_50_/mL (maximum titre, harvested from cell culture) diluted in 30 L of tank water, giving a viral titre on the immersion tanks of ~5 × 10^4^ TCID_50_/mL, on an aerated static bath for four hours. The other two groups were mock-infected with culture media. The challenge was conducted at 17 °C to allow for a slow progression of the disease following a well-established challenge model [9]. After the bath infection, each group was kept in a 600 L tank with a flow rate of 4–6 L min^−1^ for a maximum of 10 days, equivalent to 170-degree days (DD). Four control and KHV-infected carp were sampled on day four (pre-clinical fish), on day seven (fish showing mild symptoms such as loss of appetite), and on day ten (carp developing skin lesions typical of KHV) (Supplementary Figure S1). These sampling points (four, seven, and ten days) were selected to identify the presence of viral transcripts in white blood cells before a humoral response occurred [9]. Blood samples were collected from the caudal vein of MS222 (400 ppm solution, Merck, Gillingham, UK),) anaesthetised fish, placed in heparinised tubes (BD vacutainer) containing potassium ethylenediaminetetraacetic acid (EDTA) and stored at 4 °C until further use. Blood smears were left to air dry and stained with Giemsa (Sigma) following the manufacturer’s standard protocols. In parallel, kidney sections were (Merck, Gillingham, UK) collected and stored in RNAlater ^®^ (Merck, Gillingham, UK) to confirm KHV infection via real-time qPCR, as described below.

### 2.3. Leukocyte Isolation and Blood Cell Population Count

Blood leukocytes were separated using density gradient centrifugation following [20]. Briefly, equal volumes of blood and phosphate-buffered saline (PBS) (Merck, Gillingham, UK) were mixed and layered into a freshly made discontinuous (from the top layer down) 20%, 30%, 40%, and 50% density gradient of 1.130 g/mL Percoll^®^, pH 8.5–9.5 (Merck, UK) at a ratio of 1 mL of diluted blood in 6 mL. The density gradient was obtained via centrifugation at 2000× *g* for 30 min in a swinging bucket rotor Falcon 61,300 centrifuge (MSE, Cholet, France)) with the brake OFF at 4 °C. Leukocytes, mainly located between the 30 and 40% interface, were collected and washed with five volumes of PBS. Cells in PBS were split into three equal aliquots; one aliquot was pelleted via centrifugation at 500× *g* for 5 min and stored in 500 uL of RNAlater ^®^ (Merck, Gillingham, UK) at −20 °C for RNA extraction; the second aliquot was resuspended in staining buffer (eBioscience, Paisley, UK) and the size and granularity of the isolated leukocytes analysed in a flow cytometer (CytoFlex, Beckman, Wycombe, UK); and the third aliquot was used for the flow cytometry detection of KHV, as described below.

### 2.4. Flow Cytometry Detection of KHV in Peripheral Blood Leukocytes

The average size and granularity of common carp blood leukocytes were observed in a CytoFlex (Beckman, Wycombe, UK) flow cytometer. Forward scatter (FSC) and side scatter (SSC) properties of ten thousand events were measured and analysed in CytExpert version 2.3.0.84 software (Beckman, Wycombe, UK). The leukocyte population gating was conducted according to previous studies on carp [21].

To identify the percentage of KHV-positive leukocytes, approximately 10^6^ leukocytes per mL were fixed in 100 µL of IC fixation buffer (eBioscience, Paisley, UK) for 30 min at room temperature (~18–20 °C), washed with a staining buffer (eBioscience, Paisley, UK), centrifuged at 500× *g* for 5 min in an Eppendorf centrifuge 5417R (Eppendorf, Arlington, UK), and then resuspended in 500 µL of permeabilisation buffer (eBioscience, Paisley, UK) containing 1% of bovine serum albumin (BSA) (Merck, Gillingham, UK) for 15 min. A KHV monoclonal antibody (Aquatic Diagnostics, Argyll, UK) was conjugated with Alexa Flour 488 using the Zip Alexa Fluor™ Rapid Antibody Labelling Kit (Invitrogen, Gillingham, UK) per the manufacturer’s instructions. Fixed and permeabilised leukocytes were incubated with a solution containing permeabilisation buffer, 1% of BSA, and 1 µg of the Alexa Fluor-conjugated KHV antibody for 30 min in the dark at room temperature. The cells were washed twice with the staining buffer and analysed on a CytoFlex. Significant differences among groups were determined through Student’s *t*-tests for unequal variance, given that normality and equal variance assumptions for parametric testing were unmet. Data are presented as mean ± standard deviation of the mean.

### 2.5. Library Prep and RNA Sequencing

Total RNA was extracted from leukocytes and kidney sections using the Maxwell^®^ RSC simplyRNA Cells Kit or the Maxwell RSC simplyRNA Tissue Kit in the Maxwell^®^ RSC 48 extraction robot (Promega, Southampton, UK), following the manufacturer’s instructions. RNase-free DNase I (Promega, Southampton, UK) was added to remove DNA contamination during the RNA extraction, following the manufacturer’s guidelines. RNA-Seq was conducted on leukocytes harvested from control carp and KHV-infected carp sampled ten days post infection (dpi), and three replicate samples were analysed for each group. RNA quality was visualised on a TapeStation with an RNA ScreenTape & Reagents (Agilent, Cheadle, UK). Illumina-indexed libraries were prepared with concentrations ranging from 50 to 200 ng RNA with rRNA depletion as part of the lllumina Stranded Total RNA Prep Ligation with Ribo-Zero Plus protocol (Illumina, Great Abington, UK). Samples were pooled in equimolar concentrations before denaturing, diluting, and 150 paired-end (PE) sequencing on the Illumina NovaSeq 6000 platform over 0.4 lanes of an SP flow cell (Illumina, Great Abington, UK).

### 2.6. Annotation of the Common Carp Genome

The carp reference genome (ASM1834038v1; RefSeq Assembly Accession GCF_018340385.1) and corresponding protein sequences were downloaded from the RefSeq database [22]. The carp protein sequences were subjected to sequence similarity searches using Diamond v2.0.15 [23] and the full NCBI nr database (downloaded on 3 April 2022). Functional annotations were assigned to the sequences using InterProScan v5.55-88.0 and all default analysis modules [24]. All annotations were imported into OmicsBox v1.4.12 [25], which was used to add gene ontology (GO) annotations to each of the proteins (Gene Ontology Annotation database accessed on 1 August 2022; ftp://ftp.ebi.ac.uk/pub/databases/GO/goa/).

### 2.7. Sequence Pre-Processing, Read Mapping, and Abundance Estimation

Raw data quality control and the removal of adapter sequences and low-quality bases were conducted using Fastp 0.20.0 [26]. Ribosomal RNA (rRNA) sequences were removed with SortMeRNA v4.3.6 [27] using default parameters and a selection of SILVA SSU, LSU [28], and RFAM [29] databases, including rfam-5.8s-database-id98, rfam-5s-database-id98, silva-arc-16s-id95, silva-arc-23s-id98, silva-bac-16s-id90, silva-bac-23s-id98, silva-euk-18s-id95, and silva-euk-28s-id98. The nf-core/RNAseq v3.0 pipeline [30] was used to map the reads to both the common carp reference genome and the KHV genome (NC_009127.1) using STAR v2.7.6a [31] and to quantify gene expression abundances with RSEM v1.3.1 [32] and samtools v1.10 [33]. The resulting gene expression count matrices were loaded into the DEBrowser v1.18.3 [34] for differential gene expression analysis. Genes with less than one count per million in at least two samples were removed, and expression values were normalised across the samples using median ratio normalisation (MRN). Differences in gene expression between the various treatments were determined using DESeq2 v1.30.1 [35] with parametric fit dispersion, the likelihood ratio test (LRT) and the Wald test. Genes were considered differentially expressed when adjusted *p* values < 0.05 (Benjamini-Hochberg correction) and fold change (FC) > 2. Principal component analysis (PCA) plots, heatmaps (based on hierarchical clustering: Euclidean as the distance measure and complete for clustering linkage), and scatter plots of read counts were generated from within the DEBrowser using default parameters.

### 2.8. Functional Annotation Analysis

To identify enriched biological processes and pathways, gene set enrichment analysis (GSEA) was performed on a ranked list of genes, based on their associated log2 FC values (high to low), using the clusterProfiler package (v3.18.1) [36] within Rstudio v1.3.1056 [37] running R v4.0.2 [38]. Gene ontology (GO) and Kyoto Encyclopaedia of Genes and Genomes (KEGG) pathway gene sets were used for the analysis. The GO enrichment analyses were conducted using the GSEA function of clusterProfiler, with minimum and maximum gene set sizes of 10 and 500, respectively. KEGG pathway enrichment analysis was conducted using the gseKEGG function, with a minimum gene set size of 10 and the KEGG dataset for Cyprinus carpio (ccar). Processes and pathways were considered to be enriched when adjusted *p* values < 0.05 (Benjamini–Hochberg correction). Dot plots for the GO terms and KEGG pathways were generated using the dot plot function in clusterProfiler.

### 2.9. Real-Time PCR Validation of RNA-Seq Differentially Expressed Genes

Total RNA was reverse transcribed at 37 °C for 1 h in a 20 µL reaction containing 200 U of M-MLV RT, M-MLV RT 5× reaction buffer (250 mM Tris-HCl, pH 8.3; 375 mM KCl; 15 mM MgCl_2_; 50 mM DTT); 1 mM dNTP mix; 500 ng of random primers; and 25 units of RNasin^®^ Ribonuclease Inhibitor (Promega, Southampton, UK). KHV transcripts were analysed using a diagnostic real-time PCR (RT-PCR) targeting a fragment of the viral *orf90* gene [39]. The relative expression of eleven carp genes found differentially expressed (DEG) via the RNA-Seq analysis were also assayed using RT-PCR. Primers and probes were de novo designed using Primer Express v3.0 (ThermoFisher, Ringway, UK) (primers sequences in Table S1 in the Supplemental Materials). A previously described RT-PCR assay for the carp *actin beta 1* (*actb1*) gene was used as a reference gene for normalisation purposes [9,40]. Reverse-transcribed cDNA from five control and four KHV-infected carps sampled at ten dpi, which included the same RNA extracted from the samples used for generating the sequencing library, were tested with the RT-PCR assays. Each sample was analysed in duplicate in a master mix containing 2 µL of the cDNA, 500 nM of each primer, and 250 nM of the probe labelled with 6-FAM 5′ and 3′ BHQ1, in a total volume of 20 µL, by using the Taqman^®^ Universal Master Mix II with UNG (Life Technologies, Paisley, UK). PCR reactions were performed on a StepOne Plus™ Real-Time PCR System V2.3 software (Applied Biosystem, UK) with 40 cycles of PCR amplification and fluorescence detection as recommended by the manufacturer. Negative control samples containing molecular-grade water were used. PCR efficiency was calculated for each assay using serial dilutions of a control cDNA. Cycle thresholds (Ct) were used to calculate the FC (2∆∆Ct) expression of a target gene between samples using the PCR package [41] in the R Bioconductor within Rstudio v1.3.1056 running R v4.0.2 [37,38].

## 3. Results

### 3.1. Detection of KHV in White Blood Cells 

**Figure 1:**
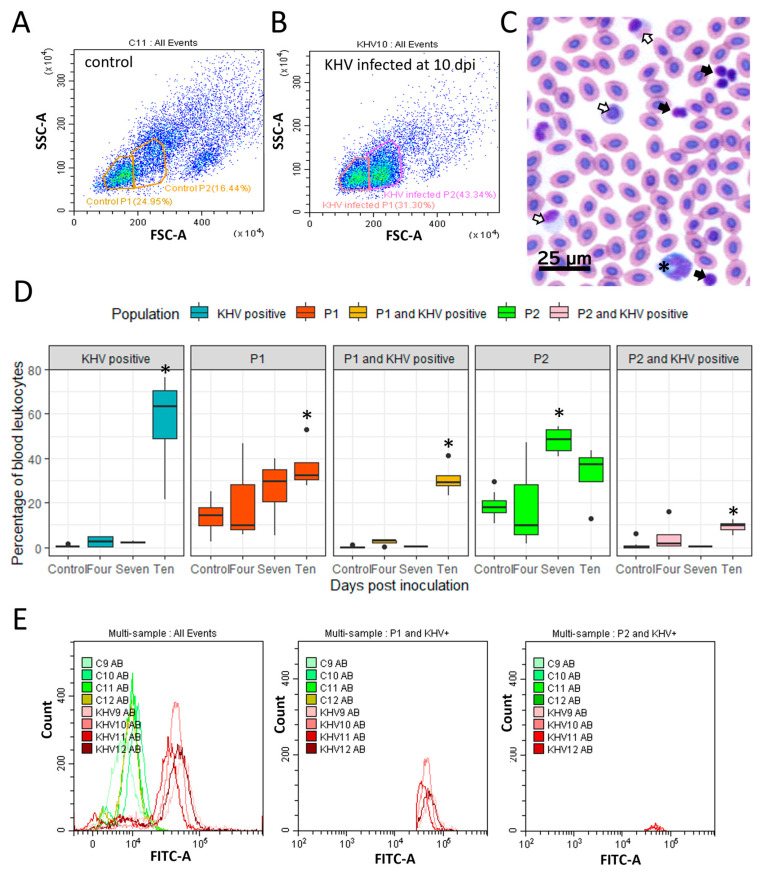
Peripheral blood leukocyte analysis in common carp infected with koi herpesvirus (KHV). (**A**,**B**) Pseudo-colour density dot plot showing the forward scatter (FSC) and side scatter (SSC) properties of blood leukocytes from (**A**) a control carp and (**B**) a KHV-infected carp sampled at ten days post infection (dpi). Two populations were gathered: P1 and P2. (**C**) Giemsa-stained blood smear photomicrograph of an infected carp at ten dpi. Black arrows: lymphocytes-like; open arrows: monocytes-like; asterisk: neutrophil-like. (**D**) Box plots showing the percentage of KHV-positive leukocytes, P1 and P2 populations in blood leukocytes, and the percentage of KHV positive cells in P1 (P1 and KHV positive) and P2 (P2 and KHV positive). Control group: 12 fish; KHV-infected group: four fish per each sampling point (four, seven, and ten dpi). Asterisk (*) denotes a significant difference (*t*-test *p*-value < 0.05) in the number of cells between control and infected carp. (**E**) Flow cytometry analysis of peripheral leukocytes of four control fish (C9, C10, C11, C12) and four KHV-infected fish (KHV9, KHV10, KHV11, KHV12) sampled at ten dpi stained with an anti-KHV antibody conjugated with Alexa Fluor 488. Leukocytes from KHV-infected carp showed increased fluorescence on the FITC channel in both P1 and P2 populations.

Flow cytometry analysis of blood leukocytes showed the enrichment of two different populations of leukocytes (P1 and P2). Based on the size and granularity properties, these populations were hypothesised to be lymphocytes (P1) and either enlarged lymphocytes or monocytes (P2) (Figure 1A,B and Figure S2). Similarly, in blood smears, an apparent higher proportion of lymphocytes and monocytes was observed in infected carp sampled at ten dpi, but this was not formally quantified (Figure 1C).

A significant increase in the number of cells representing the P1 population was observed in KHV-infected carp sampled at ten dpi, coinciding with the development of skin lesions. In contrast, for the P2 population, a significant increase in the number of cells was observed at seven dpi (Figure 1C). From the three sampling points (four, seven, and ten dpi), flow cytometry analysis identified KHV-positive leukocytes at ten dpi (Figure 1D,E and Figure S3) in both P1 and P2. The number of KHV-positive leukocytes increased to 56% ± 21% compared with the basal fluorescence reading of uninfected carp leukocytes (0.3% ± 0.5%) (Figure 1D). The detection of KHV via flow cytometry was corroborated by the detection of KHV transcripts in leukocytes at ten dpi and in kidney sections at 7 and 10 dpi by a KHV-specific Taqman RT-PCR (Supplementary Figure S4). The detection of KHV in blood leukocytes at ten dpi supported the decision to conduct the transcriptomics analysis of isolated leukocytes at ten dpi.

### 3.2. Transcriptome Mapping of Carp Blood Leukocytes

The average GC content of the sequencing data was 45.2% for the controls and 50.7% for the infected samples, and the average Q20 and Q30 scores were 98% and 94%, respectively. The raw paired reads generated for the various samples ranged from 29.64 to 34.71 million, averaging 32.28 ± 1.48 million. After the quality trimming and removal of rRNA sequences, the average number of read pairs per sample was 32.18 ± 1.47 (ranging from 29.55 to 34.58 million) and 7.35 ± 3.58 (ranging from 2.20 to 12.36 million), respectively (Supplementary Table S2). There were no significant differences between the numbers of reads for any treatment groups, both before and after quality trimming (*t*-test *p*-value > 0.05). However, the infected groups had significantly more reads than the controls after removing the rRNA reads (*p* = 0.03). The NCBI RefSeq *Cyprinus carpio* genome assembly (ASM1834038v1) consists of 43,833 genes, which represent 80,673 transcripts that encode for 80,686 protein sequences. GO annotations were assigned to 69,323 reference proteins (85.9% of the total proteins) or 38,698 reference genes (88.3%). In our study, 24,773 genes (56.5% of the total number of reference genes) or 47,482 proteins were found to be expressed across the samples, of which 21,215 genes (85.6%) had GO annotations assigned to them. A list containing all expressed genes and their annotations is presented in Supplementary Dataset S1.

### 3.3. Koi Herpesvirus Transcripts in Carp Leukocytes

We mapped the sequence reads to the KHV genome to assess viral replication in leukocytes (Supplementary Figure S5). The mapped viral transcripts represented only ~0.001% of the transcripts found in the leukocytes after read normalisation and rRNA removal. KHV transcripts found in infected blood leukocytes covered 61% (100 out of 164 KHV ORFs) of gene-encoding proteins in the viral genome. The more abundant viral transcripts were gene products of the ORF68, which encodes for a myosin-like protein; the ORF35, encoding for an unknown viral protein; the ORF92, which encodes for the major capsid protein; and the ORF62, encoding for a putative polypeptide substrate binding site. Transcripts of the viral ORF90, the target of the currently recommended RT-qPCR on the WOAH diagnostic manual [39], were expressed but were lower than those of other viral genes.

### 3.4. Hierarchical Clustering Revealed Dramatic Differential Transcription Profiles in KHV-Infected Carp Leukocytes

**Figure 2:**
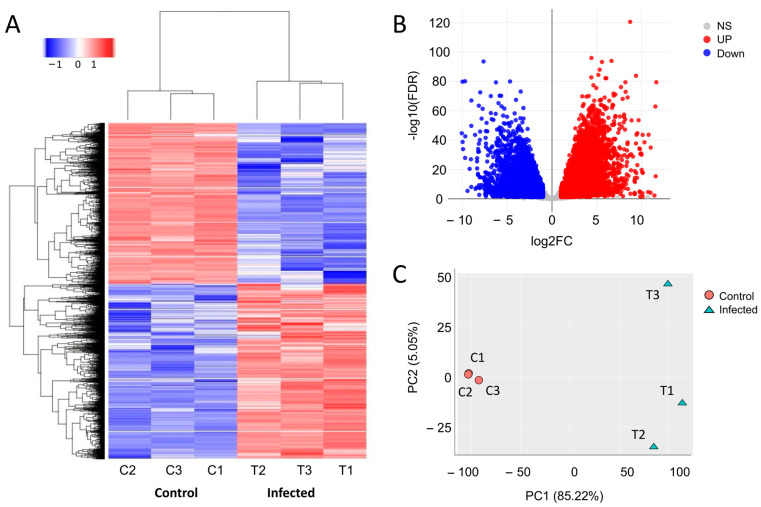
(**A**) Heatmap showing hierarchical clustering of all differentially expressed genes in common carp naïve leukocytes (samples C1, C2, and C3) and leukocytes infected with koi herpesvirus (KHV) (samples T1, T2, and T3). (**B**) Volcano plot showing up-regulated (up) and down-regulated (down) genes (*p* < 0.05, FC > 2) and genes found expressed but not significantly different from controls (NS) (*p* > 0.05). (**C**) Principal component analysis (PCA) plot of control (C1, C2, and C3) and KHV-infected (T1, T2, T3) common carp leukocytes sampled at ten days post infection.

Clustering analysis of all differentially expressed genes after quality control and normalisation revealed that KHV-infected common carp leukocytes clustered together and separated from the controls (Figure 2A). Principal component analysis (PCA) showed low replicate variability among the controls and KHV-infected, although a higher variation was observed among the KHV samples. From all the detected expressed genes, 7228 genes were found to be significantly up-regulated (*p* < 0.05, FC > 2) and 6818 genes down-regulated (*p* < 0.05, FC < 2) in the KHV-infected group (Figure 2B,C).

### 3.5. Gene Set Enrichment Analyses and Over-Representation of KEGG Pathways in KHV-Infected Leukocytes

**Figure 3:**
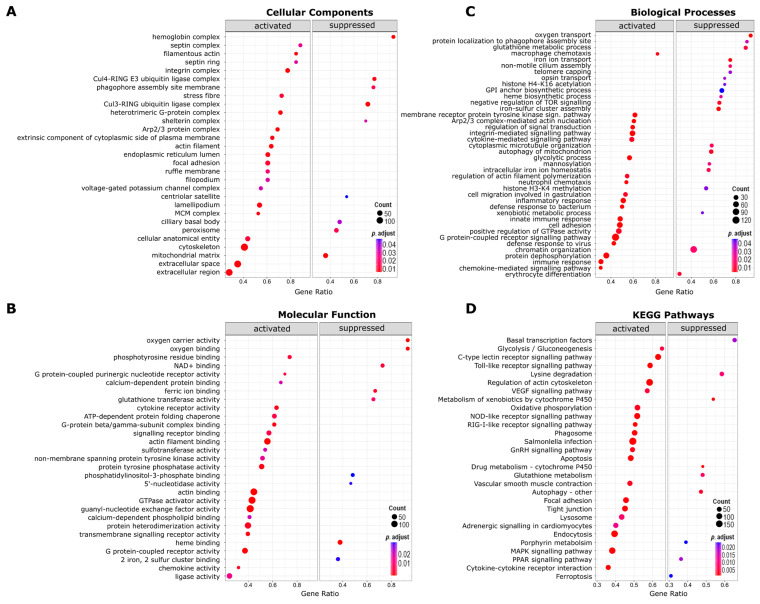
Enriched gene ontology (GO) terms and Kyoto Encyclopedia of Genes and Genomes (KEGG) pathways in Koi herpesvirus-infected common carp blood leukocytes. Enrichment of GOs belonging to (**A**) cellular components, (**B**) molecular function, (**C**) biological processes, and (**D**) enriched KEGG pathways. The size and colour of each dot represent the number of genes and adjusted *p* values, as depicted in the legend (Benjamini–Hochberg correction; FDR < 0.05), respectively.

Gene set enrichment analysis (GSEA) identified 139 unique GO terms enriched in KHV-infected leukocytes (Figure 3A–C, Supplementary Dataset S2), and 38 over-represented KEGG pathways among the differentially expressed gene lists in KHV-exposed leukocytes (Figure 3D, Supplementary Dataset S3). Examples of genes that play a crucial role in those molecular pathways and that were found differentially expressed can be found in Table 1.

Based on the list of enriched GO and KEGG pathways, our data showed that KHV infection in carp triggered strong immune and proinflammatory responses, as seen by the enrichment of numerous immune-related GO terms such as inflammatory response, innate immune response, and defence response to virus. In particular, we observed the production of pro-inflammatory chemokines and cytokines responsible for cell-to-cell communication and recognition of infection. Enriched signalling pathways included the chemokine-mediated signalling pathway and the cytokine-mediated signalling pathway, and among the KEGG pathways were the toll-like receptor (TLR) signalling pathway, C-type lectin receptor pathway, NOD-like receptor signalling pathway, and the antiviral specific RIG-I-like receptor signalling pathway. The attraction of mononuclear phagocytes (monocytes and macrophages) was deduced from a strong enrichment of the GO terms macrophage chemotaxis and neutrophil chemotaxis.

Changes in the metabolism of macrophages were suggested by the enrichment of biological processes associated with increased metabolic glycolysis, such as glycolytic process, glycolysis/gluconeogenesis, mitochondrial oxidative phosphorylation, the mitogen-activated protein kinases (MAPK) signalling pathway, and the vascular endothelial growth factor (VEGF) receptor signalling pathway. Metabolic processes observed to be suppressed included the glutathione metabolic process, glutathione transferase activity, and the KEGG pathways glutathione metabolism and peroxisome proliferator-activated receptor (PPAR) signalling pathway. Changes in the iron metabolism in KHV-exposed leukocytes were also inferred by the suppression of biological processes such as iron ion transport and intracellular iron ion homeostasis and the suppression of KEGG pathways such as ferroptosis and porphyrin metabolism.

We observed the inhibition of molecular pathways related to transcription and autophagy as seen by strong suppression of the KEGG pathway basal transcription factors and autophagy, and GO terms related to protein degradation such as Cul4-RING E3 ubiquitin ligase complex and Cul3-RING ubiquitin ligase complex. The suppression of the GO terms also suggested a disruption of epigenetic regulation in KHV-exposed leukocytes. We observed the activation of phagocytosis in KHV-infected leukocytes, aided by a strong activation of the actin cytoskeleton. Some of the molecular pathways found activated were the KEGG phagosome and regulation of actin cytoskeleton, stress fibre, filamentous actin, and regulation of actin filament polymerisation. An interesting feature from the list of enriched GO terms is the key role of septins, a component of filamentous polymers, in connecting the leukocyte’s cytoskeleton network. We observed a strong activation of septin-related GO terms, such as septin complex and septin ring, and biological processes involving septins, such as the integrin-mediated signalling pathway. We also observed activated pathways related to the extracellular trafficking of proteins, which included the Arp2/3 protein complex, extracellular space, and extracellular region. The dynamic trafficking of proteins was also supported by the activation of biological processes involved in cell signalling, enzyme activity, and protein binding, as observed by the enrichment of GO terms such as protein dephosphorylation and G-protein-coupled receptor activity.

### 3.6. Carp Blood Leukocyte Immunophenotype in Response to Koi Herpesvirus Infection

**Figure 4:**
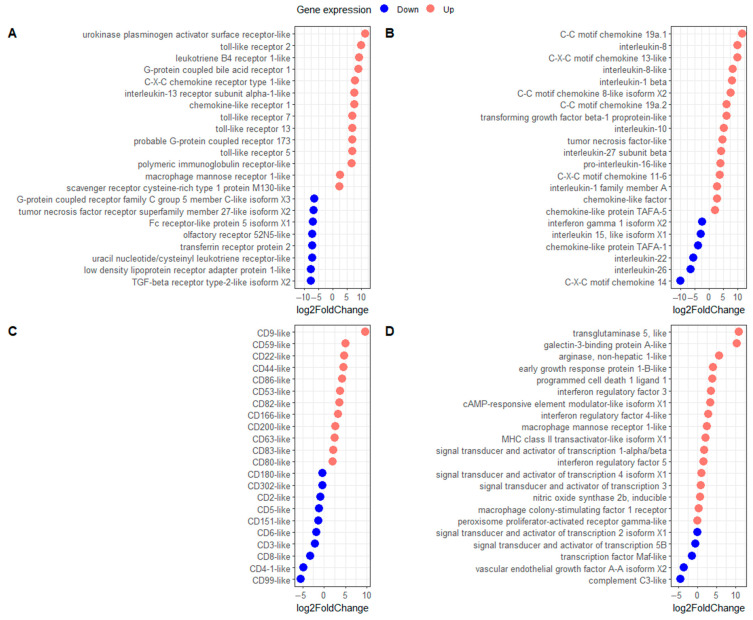
Carp macrophage phenotype in response to koi herpesvirus infection (KHV). Gene expression (log2 fold change) of either up- or down-regulated (**A**) cellular receptors, (**B**) interleukins, (**C**) cluster of differentiation (cd), and (**D**) other genes informative for macrophage phenotyping.

The gene expression of typical markers of polarised macrophages was observed in KHV-infected leukocytes (Table 1). Examples of up-regulated M2-type macrophage markers were *transglutaminase 5*-like (*tgm5l*) (Log2FC = 10.98), *arginase non hepatic 1*-like (Log2FC = 5.74), and *cAMP-responsive element modulator*-like (*crem*) (Log2FC = 3.35), while examples of M1-type markers up-regulated were *C-X-C motif chemokine 13*-like (Log2FC = 10.01) and *serum amyloid A* (Log2FC = 9.94). Interestingly, a typical marker for M1-type, *nitric oxide synthase 2b* was not differentially regulated (log2F = 0.65). Cell surface receptors found strongly up-regulated included the *urokinase plasminogen activator surface receptor*-like (*upar*) (Log2FC = 11.53), TLRs such as *tlr2* (Log2FC = 10.09), *tlr5* (Log2FC = 6.91), *tlr7* (Log2FC = 7.06), and *tlr13* (*tlr22*) (Log2FC = 7.05), several G-protein coupled receptors such as *g-protein coupled bile acid receptor 1* (*gpbar1*) (Log2FC = 9.04), the *leukotriene B4 receptor 1*-like (*btl1*) (log2FC = 9.24), a *polymeric immunoglobulin receptor*-like (Log2FC = 6.83), and the *interleukin-13 receptor subunit alpha-1*-like (Log2FC = 7.72) (Figure 4A). Up-regulated interleukins in carp leukocytes included the *tumour necrosis factor* (*tnf*)-like (Log2FC = 5.44), *transforming growth factor beta-1 proprotein*-like (Log2FC = 6.32), *il-8* (*cxcl8a*) (Log2FC = 10.08), *il1b* (Log2FC = 8.17), *il-10* (Log2FC = 5.36), and the *chemokine C-C motif chemokine 19a.1* (*ccl19a.1*) (Log2FC = 11.63). Among the interleukins observed to be down-regulated were *il-22* (Log2FC = −5.41), *il-26* (Log2FC = −6.37), and *chemokine C-X-C motif chemokine 14* (*cxcl14*) (Log2FC = −9.98) (Figure 4B). Example of CDs observed to be highly up-regulated were different isoforms of the tetraspanin *cd9* (Log2FC = 5.29) and *cd59* (Log2FC = 5.08), whether examples of CDs strongly down-regulated were *cd99* (Log2FC = −4.81), *cd8* (Log2FC = −3.18), *T-cell surface glycoprotein cd5* (Log2FC = −1.08), and *T-cell surface glycoprotein CD3 zeta chain*-like (Log2FC = −2.02) (Figure 4C). Other genes of interest for macrophage immunotyping were *galectin-3-binding protein A*-*like* (Log2FC = 10.11), interferon regulatory factors such as *irf3* (Log2FC = 3.59), *irf4* (Log2FC = 2.79), complement *c3*-like (Log2FC = −4.4), and the *vascular endothelial growth factor A-A isoform X2* (vegfaa) (Log2FC = −3.54) (Figure 4D).

### 3.7. Real-Time PCR Validation of RNA-Seq Data

**Figure 5:**
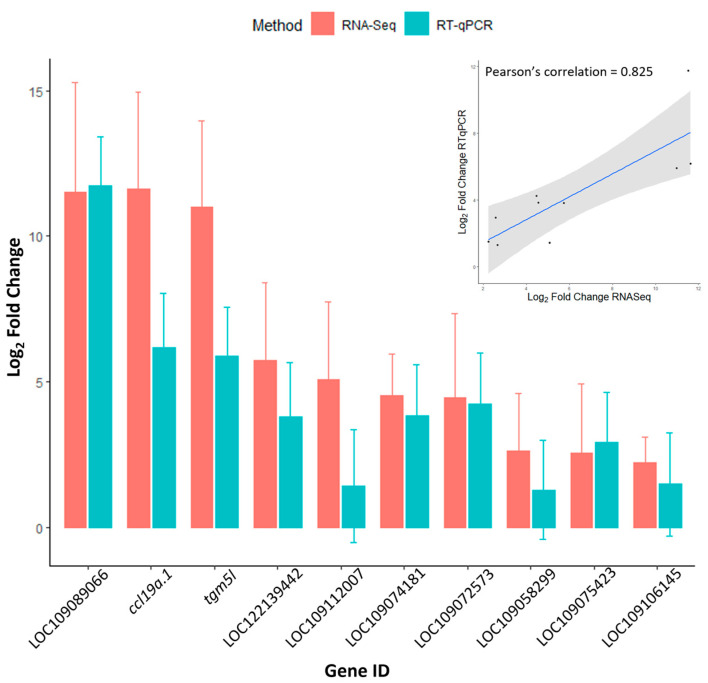
Bar plot showing a comparison of the RT-PCR and RNA-Seq fold change values obtained for ten selected genes. Error bars represent 95% confidence interval (fold change ± 1.96 × SD), number of fish were three KHV-infected fish for the RNA-Seq analysis and four KHV-infected and control carps for the RT-qPCR tests, all sampled at ten days post infection. The inset scatter plot highlights the high correlation found between both methods. Genes ID: LOC109089066 *urokinase plasminogen activator surface receptor*-like, GenBank accession number XM_042747598.1; *ccl19a.1 C-C motif chemokine 19a.1*, XM_019104238.2; *tgm5l transglutaminase 5*-like, XM042725334.1; LOC122139442 *arginase, non-hepatic 1*-like, XM_042737121.1; LOC109112007 CD59 *glycoprotein*-like, XM_019125028.2; LOC109074181 *CD44 antigen-like isoform X2*, XM_042752900.1; LOC109072573 *CD83 antigen*-like, XM_019119505.2; LOC109058299 *solute carrier family 40 member 1*, XM_042730488.1; LOC109075423 *macrophage mannose receptor 1*-like, XM_042718436.1; and LOC109106145 *allograft inflammatory factor 1-like isoform X1*, XM_019088805.2. Note the plot does not show the fold change of the carp *interleukin-10* (*il10*, XM_042734270.1) which was only detected in KHV-infected samples.

Eleven selected genes that were observed differentially expressed on the RNA-Seq data also showed significant differences (FC>2, *p*-values < 0.05) when analysed by RT-PCR. The gene expression of carp *il10* was detected by RT-PCR only in KHV-infected leukocytes. The FC direction of the genes tested via RT-PCR agreed with the RNA-Seq data. Moreover, the FC values calculated from both methods were highly correlated (Pearson’s correlation = 0.8254054) (Figure 5).

## 4. Discussion

CyHV3, or KHV, is a highly contagious virus that can cause significant mortality in carp species [1]; its eradication is challenging due to the establishment of lifelong latency in white blood cells and other tissues [7]. In this study, peripheral blood was collected during the active phase of infection, and the presence of KHV confirmed in leukocytes sampled at ten dpi. The bath immersion approach was chosen to mimic a natural route of infection [12]. KHVD outbreaks are highly influenced by water temperature, typically occurring between 16-25°C [3]. On previous experimental challenges conducted at 17°C, mortalities were recorded from day 10 to 27 with a survival rate of 40% [9]; other parameters, such as fish size/age, genetic background, and previous exposure to KHV can influence the outcome of the infection [14]. 

In this study, the detection of KHV in blood samples was accompanied by an increased number of leukocytes in the P1 and P2 populations. In a previous study, a rapid prominent leukocytosis in the blood of KHV-infected carp occurred during the initial two dpi and throughout the 14 days of a clinical infection at 25 °C [43]. Flow cytometric identification of leukocyte populations was based on previous studies on carp blood leukocytes [20]. Here, leukocytosis was observed as a significant increase in P1 at ten and P2 at seven dpi coinciding with the detection of KHV in blood at 10 dpi. P1 was believed to be lymphocytes and P2 either enlarged lymphocytes or monocytes. Enlarged lymphocytes in carp have been previously described in parasitised blood samples [44]. Currently, the identification and sorting of different subsets of carp leukocytes is difficult due to the lack of commercially available cell markers [45].

To uncover the molecular changes in carp blood leukocytes in response to KHV infection before a humoral reaction occurs, we analysed the enrichment of molecular pathways on unsorted blood leukocytes. The RNA-Seq analysis of carp blood leukocytes showed clear differences in the gene expression profile between uninfected and KHV-infected samples. However, a higher inter-group variation was observed among the infected samples, which might be attributed to differences in the level of infection or in host response. We confirmed the presence of KHV in leukocytes sampled at ten dpi and estimated that, on average, 56% ± 21% of the blood leukocytes were KHV-positive in the infected group. The variation in KHV-positive leukocytes within the infected samples might explain the inter-group variability observed in the RNA-Seq data. 

The analysis of the RNA-Seq data showed low levels of viral transcripts in the KHV-infected samples, with only 61% of the KHV ORFs identified. The RNA extraction and sequencing method used in this study was optimised to study the host response, and thus a specific enrichment for viral transcripts was not undertaken during the library preparation. Moreover, the presence of uninfected leukocytes in the bulk RNA might jeopardise the sequencing of low-expressed viral transcripts [46]. Similar to other herpesviruses, the expression of KHV genes is classified into three groups of genes: immediate–early IE, early E, and late L [47]. The top 15 KHV transcribed genes belonged to the E and L groups. Two major antigenic proteins, the ORF 62 encoding for a putative polypeptide substrate binding site and the ORF 68 encoding for a myosin-like protein [48], and a structural protein, the ORF92 encoding for the major capsid protein [49], were among the top five most abundant KHV transcripts found in carp leukocytes. The viral ORF6, identified as the primary viral transcript expressed [50] and translated [51] in KHV-infected IgM+ B cells during latency, was ten times lower than those top-expressed viral genes.

KHV infection triggered the recruitment of leukocytes via TLR and C-type lectin signalling. The molecular analysis suggested that KHV triggers a robust immune response, which is mediated by pattern recognition receptors (PRRs), mainly TLRs and C-type lectin stimulation, with the activation of several signalling pathways involved in the immune response and antiviral responses, consistent with that reported in the literature [8]. Our data also suggested that KHV infection attracted macrophages which is in agreement with published data noting a higher presence of T-cells and macrophages in KHV-susceptible carps [14]. Interestingly, we found that the genes *tlr2*, *tlr5*, *tlr7,* and *tlr13* were highly expressed in KHV-exposed leukocytes, suggesting that KHV proteins may act as ligands of those receptors. Similarly, the gene expression of *tlr2*, *tlr5*, and *tlr7* was previously found up-regulated in the spleen of KHV-infected carp [8] strongly suggesting an important role in recognising KHV proteins. Little is known about TLR ligands in fish species despite their importance in initiating immune responses. The carp *tlr2* is an orthologue of the mammalian TLR2 with lipopeptides acting as its ligand [52], and it has been associated with immunosuppression and M2-type macrophages [53]. *Tlr13* and the soluble form of *tlr5* are so far described exclusively in teleost species [54,55]. In some fish species, flagellin is a ligand for soluble *tlr5* [56], while the teleost ligand *tlr7* is still unknown despite its orthologous in mammals playing an essential role in antiviral immunity [56].

Among the activated PRRs, the cellular receptor that showed the higher up-regulation in KHV-exposed blood leukocytes was an *upar*-like gene (log2FC > 10). High blood protein levels of their soluble form have been linked to immunosenescence and systemic chronic inflammation [57]. Interestingly, in human macrophages, the uPAR system participates in the maintenance of intra-cytoplasmic vesicles that actively accumulate virions, thus sustaining the presence of viral reservoirs of macrophage origin [58]. Other PPRs that were highly up-regulated were galectin proteins, such as *galectin 2*-like and *galectin-3*-binding proteins, and lipid mediators or G-protein-coupled receptors, such as *blt1*. Galectin family proteins have been previously described as involved in immune responses in different species of koi and glass carp [59,60]; in particular, the genes expression of *galectin-3* was observed up-regulated in the kidney of KHV-infected carp [12], and it has been associated with KHV-resistant common carp [11]. The expression *btl1* has been shown to activate several kinases and downstream immune signalling pathways and play a role in both acute and chronic inflammation [61]. Thus, our dataset has identified specific PRRs activated in KHV-exposed leukocytes, in particular glycan-binding proteins and TLRs, and the subsequent initiation of specific signalling pathways that can aid in our understanding of carp innate immune responses to KHV and other cyprinid herpesviruses. Following KHV-induced inflammation, activation of the interferon (IFN)-I system has been described in KHV infection [10,62]. Similarly, we observed increased gene expression of an array of interferon-stimulated genes (ISG) in KHV-infected leukocytes, and amongst the antiviral proteins, the *interferon-induced protein 44*-like showed higher up-regulation. This gene has been observed highly up-regulated in virally infected zebrafish models [63]; thus, it might be a useful marker for studying interferon-induced antiviral responses in cyprinid species.

Enriched molecular pathways suggest macrophage polarisation in KHV-infected carp. Macrophage polarisation states range from classically activated “pro-inflammatory” M1 macrophages on one extreme to activated “anti-inflammatory or regulatory” M2 macrophages on the opposite extreme [64]. After PPR stimulation, macrophage activation and differentiation typically occur through changes in cell metabolism [64]. In this regard, we observed a marked increase in both metabolic glycolysis and oxidative phosphorylation (OXPHOS) and the activation of MAPK and VEGF signalling pathways. The enrichment of these transduction pathways were previously noted in the spleen of KHV-infected carps [8] and in orange-spotted grouper (*Epinephelus coioides*) infected with Singapore grouper iridovirus (SGIV) [65]. The activation of the MAPK signalling pathway contributes to pro-inflammatory cytokine production, and it has been linked to enhanced virus replication [66]. We also observed that the glutathione metabolism and the PPAR signalling pathway decreased in KHV-exposed leukocytes. In mammals, M1 macrophages are typically characterised by glycolytic metabolism and the expression of inducible nitric oxide synthase (iNOS), reactive nitrogen intermediates, and reactive oxygen species (ROS), promoting Th1 responses and strong microbicidal activity. Conversely, M2 macrophages are characterised by increased phagocytic activity and mitochondrial respiration, with a high expression of scavenging, mannose, and galactose receptors through the arginase pathway [67]. Interestingly, our data show that KHV-exposed leukocytes exhibited increased mitochondrial respiration through activation of the OXPHOS pathway, low production of ROS compared with naïve leukocytes, low glutathione metabolism, and the up-regulation of *arginase non-hepatic 1*-like, *macrophage mannose receptor-1*, and scavenger receptors. We also observed up-regulation of the gene expression of *crem* and *il-13* receptors and down-regulation of *ifng1*. In fish, macrophage polarisation towards M1- and M2-like states has been previously demonstrated [68,69]. In common carp, macrophage polarization of kidney-derived macrophages showed differential activity of inducible NO synthase and arginase depending on the type of stimulation [70]. In particular, carp macrophage polarisation into an M2-type phenotype was driven by *il-4/13* or *cAMP* stimulation [42].

Interestingly, we found an up-regulation of *il-10* in KHV-exposed leukocytes. IL10 is a typical marker of M2 macrophages [64], and in carp, it has been shown to promote anti-inflammatory activities on phagocytes [71]. We also observed a robust up-regulation of transglutaminase proteins, a conserved marker for mammalian M2 macrophages [72], which has been previously found to be expressed in carp M2 macrophages [42]. Among the cytokines, we observed the gene up-regulation of both pro-inflammatory cytokines, such as *tnf*-like, and anti-inflammatory cytokines and chemokines, such as *tgfb1* and *il-10*, characteristic of an M2 type [64]. In addition, we observed a strong up-regulation of *cxcl8/il8*, which has been shown to attract neutrophils in carp [73], and *ccl19a.1*, a chemokine that attracts T-cells and macrophages and that has been linked to carp susceptibility to KHV [14]. Our dataset also showed significant changes in the regulation of iron metabolism in carp leukocytes. In particular, we observed the down-regulation of *ferritin*-related genes, which relates to low iron storage, and high expression of *ferroportin*, which suggests high iron export, suggesting that KHV-exposed leukocytes are set in an iron-export mode which correlates with typical M2 polarised macrophages [67]. Based on the current knowledge on herpesviruses, the balance of M1 and M2 macrophages play a role in viral clearance and latency [74]. Overall, our dataset suggests both pro- and anti-inflammatory responses in KHV-infected carps. This finding and its impact on KHV pathogenicity require further investigation.

Evidence of altered epigenetic and transcriptional regulation in carp leukocytes. Interestingly, we observed a strong suppression of epigenetics and regulation of transcription pathways, such as histone H3-K4 methylation. Recent studies have shown the important role of histone-modifying enzymes, particularly histone acetyltransferases (HATS) and histone methyltransferases (HMTs), in regulating macrophage function. However, the exact role of epigenetic regulation on inflammatory processes is not yet well understood [75]. Remarkably, the inhibition of H3-K4 methylation has been shown to compromise immune responses in murine macrophages [76]. Taken together, the down-regulation of histone H3-K4 methylation and other critical epigenetic regulation in KHV-exposed leukocytes might suggest an immunocompromised response [77]. KHV establishes a long-lasting life infection through latency with reactivation episodes and the appearance of secondary infections [7]. The disruption of epigenetic regulation may have contributed towards immunosuppression associated with chronic KHV infection in carp. In grass carp, epigenetic mechanisms such as DNA methylation have been linked to immune responses and carp susceptibility to viral infection [78]. The role of epigenetic regulation on carp inflammatory responses requires further research.

High phagocytosis but low autophagy of KHV-infected leukocytes might support KHV latency. We observed a strong regulation of phagocytosis supported by a hyperactive rearrangement of the actin cytoskeleton, extracellular matrix, and numerous molecular pathways denoting high macrophage mobility, with up-regulation of Rho GTPase activator enzymes, G-protein-coupled receptors, and protein phosphatases [79]. Among the carp genes highly up-regulated were *allograft inflammatory factor 1*-like, which has been associated with enhanced macrophage phagocytic activity in mice [80], the macrophage *mannose receptor 1*-like, previously proposed as a marker of carp M2 macrophages [42], numerous septin genes involved in molecular pathways of the leukocyte’s cytoskeleton network, and several isoforms of the tetraspanin *cd9* which play an essential role in activating the leukocyte’s motility and adhesion [81].

Our dataset also showed molecular evidence of low autophagy activity in KHV-exposed leukocytes, suggesting poor clearance of phagocytised viral particles. Indeed, the activation of autophagy has been observed in resistant carp families to KHVD [11]. Several mammal herpesviruses have developed strategies to escape the autophagy of immune cells either by expressing specific anti-autophagic proteins or hijacking autophagy [82]. Recent studies suggest a role of the complement system in autophagy [83]. In the present study, the activation of complement pathways was not observed. An increase in complement haemolytic activity in blood plasma [62] as well as C-reactive proteins in infected tissues such as the liver, spleen, and kidney [84] have been described in KHV infection. However, the gene expression of *c3*, a central component of the complement system, was not observed at mucosal sites, such as gills and skin [9]. Similarly, cyprinid herpesvirus 2 (CyHV2) infection in gibel carp (*Carassius auratus* gibelio) induced the up-regulation of *c3* transcription in internal organs but not at mucosal sites [85].The role of the complement system and autophagy in clearing KHV infection requires further research.

Although this study has provided valuable data on carp immune responses against KHV infection, the mechanisms by which KHV infects and establishes latency in blood leukocytes, mainly B cells, are still to be elucidated. Our RNA-Seq sequencing approach used pooled isolated leucocytes and therefore did not provide data on transcription for individual cell types. Additionally, the risk of some contamination (with erythrocytes, for example) during the process of isolation of leucocytes cannot be excluded. Future work on identifying specific subsets of blood leukocytes in KHV-infected carp should involve either cell sorting if antibodies were available or single-cell sequencing [86]. To facilitate this, we suggest genes that could be developed to be used as cellular markers for further characterisation and cell sorting, such as *cd83*, *cd59*, and *cd44*. In mammals, CD83 is induced explicitly in IL-4-stimulated M2-type macrophages [87], CD44 has been shown to promote the accumulation of M2 macrophages [88], and CD59 prevents complement activation by inhibiting the formation of the membrane attack complex [88] These markers require validation prior to use in cell sorting studies. Interestingly, our data also showed down-regulation of cytotoxic T cell markers, such as cd8, *cd5*, and *cd3*, which suggests a T cell dysregulation in KHV-infected carps likely mediated by the up-regulation of *il10*, an anti-inflammatory cytokine [71], which could contribute to the lack of clearance of KHV. The role of cytotoxic T cells in KHV chronic infection and latency requires further research.

## 5. Conclusions

This study describes the molecular responses to KHV in carp blood leucocytes following infection at a relatively low temperature (17 °C). We identified several PRRs and cytokines involved in KHV recognition and immune signalling. We suggested candidate genes that can be used for further characterisation of carp leukocytes following viral infection. We also identified molecular pathways supporting macrophage polarisation in blood leukocytes exposed to KHV, characterised by high phagocytosis, low autophagy, and increased iron export. Further work is required to fully characterise the response to KHV in different leukocyte subsets and the molecular events leading to the establishment of KHV latency. Overall, this work contributes to a better understanding of the innate immune response to herpesvirus infection in cyprinids. Our results point towards new research priorities on understanding the response to KHV at the single-cell level and open new avenues for the development of treatments in the future.

## Figures and Tables

**Table 1:** List of selected differentially expressed genes (*p*-values < 0.05) in koi herpesvirus-infected common carp blood leukocytes. The full list of genes can be found in Supplementary Dataset S1. The number of reads (transcripts) per million (TPM) mapped reads is shown for each sample. FC = fold change in control versus KHV-infected leukocytes. C: control samples; KHV: KHV-infected carp blood leukocytes. * Carp macrophage polarisation signatures according to Wentzel et al., 2020 [42].

Gene Accession	C (1)	C (2)	C (3)	KHV (1)	KHV (2)	KHV (3)	log2FC	Gene
Innate immune response								
XM_042741157.1	0	0	0	62.82	25.63	346.84	10.09	*toll-like receptor 2*
XM_042711149.1	1.97	8.18	7.71	570.11	636.4	1111.85	*7.05*	*toll-like receptor 13*
XM_042778173.1	17.08	12.65	6.17	1927.08	1064.39	1366.38	6.91	*toll-like receptor 5*
XM_042764380.1	4.6	0.74	0.77	180.61	298.7	372.01	7.06	*toll-like receptor 7*
XM_042710956.1	24.97	41.66	17.72	4765.08	2668.21	3978.87	7.08	*complement factor B*-like
XM_042750556.1	5.91	2.98	6.17	293.69	98.08	275.51	5.46	*antiviral innate immune response receptor RIG-I*-like
XM_042713940.1	103.16	107.86	145.65	2461.07	2047.41	2216.7	4.24	*antiviral innate immune response receptor RIG-I*
XM_042772231.1	26.94	19.34	16.18	1204.62	560.61	966.4	5.44	*tumour necrosis factor receptor superfamily member 1A*
XM_019090552.2	0	0	0	20.42	10.03	20.98	7	*interferon-induced protein with tetratricopeptide repeats 5*-like
XM_042724080.1	21.02	14.87	23.89	243.43	91.39	271.31	3.38	*grass carp reovirus (GCRV)-induced gene 2o*
XM_019104238.2	4.6	1.49	0	8259.58	4073.65	7973.12	11.63	*C-C motif chemokine 19a.1*
XM_019081222.2	7.225.2	5.20	10.78	1367.95	1747.60	1747.60	8.04	*interferon-induced GTP-binding protein MxB-like*
XM_042768955.1	0	0	0	111.51	14.49	117.48	9.25	*interferon-induced protein 44-like isoform X1*
XM_042747598.1	17.08	14.13	18.5	67491.73	18,406.65	61,035.41	11.53	*urokinase plasminogen activator surface receptor*-like
XM_019072681.2	0	2.98	0	144.49	71.33	114.68	6.83	*polymeric immunoglobulin receptor*-like
XM_042759636.1	1.97	0	0	161.77	37.89	246.14	7.72	*interleukin-13 receptor subunit alpha-1*-like
XM_019084892.2	58.48	51.33	48.55	675.34	624.14	609.77	3.59	*interferon regulatory factor 3*
XM_042746991.1	231.93	215.72	237.36	1397.8	1259.43	2093.63	2.79	*interferon regulatory factor 4*-like
XM_042711994.1	7.88	11.9	10.79	0	1.11	0	−4.4	*complement C3*-like
XM_019097650.2	45.99	88.52	73.21	3.14	12.26	1.4	−3.54	*vascular endothelial growth factor A-A isoform X2*
XM_019068368.2	11.17	9.67	16.18	1.57	3.34	8.39	−1.47	*transcription factor Maf-like*
Attraction of mononuclear phagocytes								
XM_019080073.2	14.45	23.06	5.39	5500.11	3708.08	3164.91	8.17	*interleukin-1 beta*
XM_042733011.1	413.28	678.41	315.19	26,566.05	17,916.25	16,020.36	5.43	*collagenase 3a*
XM_019074825.2	93.96	125.71	91.71	2272.6	1176.96	1724.41	4.05	*ras-related C3 botulinum toxin substrate 2*
XM_019099675.2	0	0	0	175.9	215.11	40.56	10.08	*interleukin-8*
XM_042763296.1	0	0.74	0	86.38	32.32	46.15	7.72	*tumour necrosis factor ligand superfamily member 11 isoform X1*
XM_042744022.1	0	0.74	0	257.57	53.5	160.83	9.24	*leukotriene B4 receptor 1*-like
Mitochondrial and iron metabolism								
XM_042767134.1	4.6	2.23	6.17	650.21	283.09	351.04	6.63	*fructose-bisphosphate aldolase A*-like
XM_042772236.1	0.66	0	0	28.27	0	13.99	5.75	*glyceraldehyde-3-phosphate dehydrogenase*
XM_042758635.1	126.81	52.81	129.47	518.29	779.06	949.61	2.86	*vascular endothelial growth factor C*-like
XM_042729971.1	264.79	237.29	190.35	87.95	81.36	86.71	−1.44	*hydroxyacylglutathione hydrolase, mitochondrial isoform X1*
XM_042768274.1	13.8	5.95	10.02	0	1.11	0	−4.36	*5-oxoprolinase*
XM_019069346.2	2540.11	2938.27	2714.99	4.71	34.55	8.39	−7.36	*microsomal glutathione S-transferase 1*
XM_042714822.1	15.77	11.16	6.94	237.15	112.57	303.48	4.26	*cytochrome c oxidase subunit 6B1-like*
XM_042713191.1	3.29	2.23	6.17	80.1	37.89	67.13	3.99	*succinate dehydrogenase [ubiquinone] iron-sulphur subunit, mitochondrial*-like
XM_019067779.2	201.05	278.95	181.87	3.14	1.11	0	−7.36	*ferritin, lower subunit*-like
XM_042720263.1	3.29	46.86	53.95	0	0	0	−7.13	*ferritin, middle subunit*-like
XM_042777542.1	316.69	288.62	220.41	1.57	11.15	12.59	−4.97	*glutaredoxin-related protein 5, mitochondrial*-like
XM_042730488.1	1854.81	1651.38	1301.62	10,618.57	5679.7	13,834.43	2.65	*solute carrier family 40 member 1*
Autophagy and protein degradation								
XM_019066281.2	56.51	60.25	68.59	4.71	8.92	1.4	−3.56	*BTB/POZ domain-containing adapter for CUL3-mediated RhoA degradation protein 1*-like
XM_042763993.1	21.03	14.13	15.41	0	1.11	0	−5.13	*FUN14 domain-containing protein 1*-like
XM_042714562.1	74.25	75.13	81.69	7.85	8.92	2.8	−3.54	*sorting nexin-7-like isoform X2*
Epigenetic and transcriptional regulation								
XM_019116784.2	9.86	5.95	11.56	0	0	0	−5.2	sclerostin domain-containing protein 1a
XM_042722790.1	239.16	196.38	201.91	7.85	14.49	5.59	−4.46	C2H2-type zinc finger protein
XM_042767739.1	204.34	190.43	171.85	36.12	49.04	54.54	−2.01	male-specific lethal 1 homolog
XM_042729995.1	19.05	17.11	17.72	4.71	0	0	−3.69	histone-lysine N-methyltransferase SETD1B-like isoform X2
Phagocytosis and actin network								
XM_042742454.1	0	1.49	0	48.69	14.49	227.96	7.63	*spectrin beta chain, non-erythrocytic 5 isoform X1*
XM_019088805.2	26.94	24.55	21.58	331.39	254.12	1026.53	4.46	*allograft inflammatory factor 1-like isoform X1*
XM_042737753.1	0.66	0	0	15.71	24.52	12.59	6.1	*smoothelin-like 1 isoform X1*
XM_042718436.1	25.62	23.06	20.04	207.31	177.21	22.38	2.56	*macrophage mannose receptor 1*-like
XM_042772413.1	53.88	43.14	70.9	373.79	345.51	346.84	2.67	*septin-2B isoform X2*
XM_042725715.1	34.17	39.42	35.45	480.59	558.39	383.2	3.71	*septin 9b isoform X1*
XM_042732942.1	5.26	0.74	7.71	36.12	25.63	64.33	3.2	*septin-9*-like
Extracellular trafficking of proteins								
XM_019107912.2	2.63	1.49	0	1444.92	503.77	991.57	9.42	*galectin-2*-like
XM_042731380.1	0	1.49	0	739.73	292.01	567.81	10.11	*galectin-3-binding protein A*-like
XM_042734258.1	11.17	15.62	11.56	1176.35	499.31	2032.09	6.6	*SLIT-ROBO Rho GTPase-activating protein 2 isoform X1*
XM_042726165.1	0	0	0	26.7	8.92	27.97	7.31	*rho guanine nucleotide exchange factor 25*
XM_042727426.1	1.31	0	0	119.36	25.63	247.54	8.11	*serine/threonine-protein phosphatase 2A 56 kDa regulatory subunit beta isoform*
XM_042764581.1	0	0	0	53.4	22.29	54.54	8.34	*tyrosine-protein kinase transmembrane receptor ROR2*-like
XM_042764258.1	0.66	0	0.77	424.05	179.44	155.24	9.04	*G-protein coupled bile acid receptor 1*
XM_019081835.2	0	0	0	39.26	15.6	26.57	7.66	*psychosine receptor*-like
XM_042717793.1	39.42	55.05	8.48	3726.94	2631.43	1974.75	6.34	*epidermal growth factor receptor*-like
Interleukins								
XM_019113158.2	1.31	0.74	1.54	42.41	43.47	22.38	4.91	*tumour necrosis factor*-like
XM_019116350.2	3.29	5.21	8.48	560.69	270.83	507.67	6.32	*transforming growth factor beta-1 proprotein*-like
XM_042734270.1	0.66	0.74	0	29.84	10.03	19.58	5.36	*interleukin-10*
XM_042721949.1	11.83	8.93	10.79	0	0	0	−5.41	*interleukin-22*
XM_042721950.1	34.17	14.88	12.33	0	0	0	−6.37	*interleukin-26*
XM_019098159.2	1081.49	851.73	756.78	1.57	1.11	0	−9.98	*C-X-C motif chemokine 14*
XM_042721947.1	7.88	6.69	10.79	1.57	3.34	0	−2.3	*interferon gamma 1 isoform X2*
Cluster of differentiation								
XM_019082792.2	34.82	54.3	36.99	2091.99	1338.56	1496.45	5.29	*CD9 antigen isoform X1*
XM_042721119.1	1.31	2.23	0.77	1551.71	626.37	1288.06	9.65	*CD9 antigen*-like
XM_019125028.2	103.16	122.74	81.69	5396.45	2833.16	2206.91	5.08	*CD59 glycoprotein-like*
XM_042765175.1	159	87.03	107.12	1380.52	802.47	1314.63	3.31	*CD166 antigen homolog isoform X2*
XM_042716436.1	804.22	794.45	945.58	18.85	44.58	25.17	−4.81	*CD99 molecule*
XM_042778750.1	24.31	17.11	31.6	3.14	2.23	2.8	−3.18	*T-cell surface glycoprotein CD8 alpha chain*
XM_019112429.2	17.74	8.92	15.41	1.57	5.57	12.58	−1.08	*T-cell surface glycoprotein CD5*
XM_019075258.2	63.07	87.03	53.17	9.42	26.74	12.58	−2.02	*T-cell surface glycoprotein CD3 zeta chain*-like
Carp M1-type macrophage *								
XM_019114762.2	0	0	0	138.21	51.27	222.37	10.01	*C-X-C motif chemokine 13*-like
XM_042759493.1	5.91	14.88	8.48	6129.9	3212.11	19460.79	9.95	*serum amyloid A*
XM_042731330.1	0.65	2.23	0	40.83	60.18	40.55	5.62	*cis-aconitate decarboxylase*-like
XM_019068715.2	15.76	10.41	4.62	284.27	21.17	258.73	4.18	*olfactomedin-4*-like
Carp M2-type macrophage *								
XM_042725334.1	0	0	0	496.3	24.52	288.1	10.99	transglutaminase 5, like
XM_042737121.1	72.93	83.31	29.28	5699.57	3166.41	1065.69	5.74	arginase, non-hepatic 1-like
XM_019101597.2	6.57	2.97	5.39	207.31	140.43	180.41	5.12	*metalloreductase steap4*
XM_019074086.2	132.72	38.68	87.08	1157.50	863.76	1064.29	3.57	*heparin-binding EGF-like growth factor a*
XM_042714579.1	44.68	43.89	59.34	713.03	590.71	209.78	3.36	*cAMP-responsive element modulator-like isoform X1*
XM_042777120.1	97.24	26.77	54.71	238.72	317.64	339.84	2.32	*angiopoietin-1*-like
XM_042777566.1	76.21	36.44	47.00	194.74	215.10	290.89	2.12	*phospholipid phosphatase 3-like isoform X1*

## Data Availability

The data have been deposited with links to BioProject accession number PRJNA1006656 in the NCBI BioProject database (https://www.ncbi.nlm.nih.gov/bioproject/), accessed om 27 February 2024.

## Supplementary Materials

The following supporting information can be downloaded at: https://www.mdpi.com/article/10.3390/v16030380/s1, Figure S1: Skin gross pathology of common carp experimentally infected with koi herpesvirus (KHV) at 17 °C. (A) control carp and (B,C) KHV-infected carp. Circles denote skin lesions; Figure S2: Forward scatter (FSC) and side scatter (SSC) properties of common carp blood leukocytes. Pseudo-colour density dot plots of control fish (left) and koi herpesvirus (KHV)-infected leukocytes (right); Figure S3: Flow cytometry analysis of blood leukocytes from common carp experimentally infected with koi herpesvirus (KHV). An anti-KHV antibody conjugated with Alexa Fluor 488 was used to gather infected cells—pseudo-colour dot plots of control (left) and KHV-infected leukocytes (right). A shift of the leukocyte population towards an increase in the number of KHV-positive cells was observed; Figure S4: Box plot showing the relative gene expression of koi herpesvirus (KHV) orf90 gene in common carp white blood cells (WBCs) and kidney sections taken at 4-, 7-, and 10 days post challenge; Figure S5: Pie chart showing the transcript abundance of 100 viral protein-coding genes expressed in carp blood leukocytes; Table S1: Summary of the common carp genes and the nucleotide sequences of primers and probes used for RT-qPCR assays; Table S2: The number of Illumina read sequences obtained before and after quality trimming and rRNA removal; Dataset S1: List of differentially expressed genes in koi herpesvirus-infected common carp blood leukocytes; Dataset S2: List of GO terms enriched on koi herpesvirus-infected common carp blood leukocytes; Dataset S3: List of KEGG pathways enriched on koi herpesvirus-infected common carp blood leukocytes.
