# Supplementary material for: Transcriptomic Responses to Koi Herpesvirus in Isolated Blood Leukocytes from Infected Common Carp
Source: Viruses. 2024 Feb 28;16(3):380. doi: 10.3390/v16030380 (PMC10974277; doi:10.3390/v16030380)
Supplement: Supplementary file 1 [file viruses-16-00380-s001.zip › Supplement Table S1_final.pdf]

Supplement Table S1. Summary of the common carp *Cyprinus carpio* genes and the nucleotide sequences of the primers and probes used for the RT-qPCR assays.

GenBank accession no.	Gene name	Gene symbol/ID	Forward primer (5' → 3')	Reverse primer (5' → 3')	Probe (Fam 5'→ MGB 3')	qPCR efficiency (%)	Function
XM_042747598.1	*urokinase plasminogen activator surface receptor-like*	LOC109072973	TGTTGAGCTGTACAGGGAATGAA	GCCACCGAAAGTCCCTGTT	CCGCTGCTTGAAAG	92.7	Pattern recognition cell surface receptor
XM_019125028.2	*cd59 glycoprotein-like*	LOC109112007	CTGTGTCCCAGAGAAAGCAGAA	CCGGTGGGCAGGTTACAGT	CCTGCGAGATCAC	136.8	Antigen cluster of differentiation
XM_042752900.1	*cd44 antigen-like isoform X2*	LOC109074181	CGTGCAGGTACGGATGGATA	TGTGGCGTGTGACGGAGTA	ACAGCTCAGAGGTGGTG	107.2	Antigen cluster of differentiation
XM_042737121.1	*arginase, non-hepatic 1-like*	LOC122139442	GGACGCTCACGCAGATATCA	GGCCGTGGAGGTTTCCA	CACTCCTCTGACGTCAC	105.3	M2-type macrophage marker
XM_042734270.1	*interleukin-10*	LOC109076801	CAGTGCGCAGTGCAGAAGAG	CCCTCCACAAATGAGCAACA	CGACTGCAAGACTG	-	Anti-inflammatory cytokine
XM_042730488.1	*ferroportin or solute carrier family 40 member 1*	LOC109058299	AACCGCTGAGGTGAAGAAAAAC	GTGCGGATGGGTTCTGTCAT	CCAGTTGCTGCTACCA	121.2	Involved in cellular iron metabolism
XM_042725334.1	*transglutaminase 5, like*	*tgm5l*	TGTTAAAGCCGTTTATGAGCAGAA	GCGTAAACGAACGGCACAT	TGGACGCGCAATAC	95.2	M2-type macrophage marker
XM_042718436.1	*macrophage mannose receptor 1-like*	LOC109075423	AGCCGAGTCACAGTGCTGTCT	CACTTTCCCTCCTCTCCTCTCA	TCCAGAGGACTGTGTGTTA	111.1	M2-type macrophage marker
XM_019119505.2	*CD83 antigen-like*	LOC109106145	TTGCGCACAGGAGATGGA	CAGCCGATGGTCCTCTTCAG	AGTCCACAGAGCTTG	90.5	M2-type macrophage marker

XM_019104238.2	*C-C motif chemokine 19a.1*	*ccl19a.1*	CGGAGGCTCAGGCAGACTT	GGATGGCTTTGTGGCTAACTG	CAATGGACTGTTGCCTG	98.4	Pro-inflammatory cytokine
XM_019088805.2	*allograft inflammatory factor 1-like isoform X1*	LOC109072573	GCGAGCGCTCACACAACA	GGTCGAGAGGAGGCGATGA	CAGTGTCTCTGTTCTCTAC	110.1	Macrophage phagocytic activity
AJ438158.1	*actin beta 1*	*actb*	CACCATGTACCCTGGCATTG	GAGGGAGCAAGGGAGGTGAT	TGACCGTATGCAGAAGG	88.2	Reference gene used for normalization purposes [9]
AF411803	Koi herpesvirus *orf90*	KHV *orf90*	GACGCCGGAGACCTTGTG	CGGGTTCTTATTTTTGTCCTTGTT	CTTCCTCTGCTCGGCGAGCACG	-	KHV WOAH-recommended diagnostic RT-qPCR [39]

References

[9] I. Cano, B. Mulhearn, S. Akter, R. Paley, Seroconversion and skin mucosal parameters during koi herpesvirus shedding in common carp, Cyprinus carpio, Int. J. Mol. Sci. (2020). https://doi.org/10.3390/ijms21228482.

[39] O. Gilad, S. Yun, F.J. Zagmutt-Vergara, C.M. Leutenegger, H. Bercovier, R.P. Hedrick, Concentrations of a Koi herpesvirus (KHV) in tissues of experimentally infected Cyprinus carpio koi as assessed by real-time TaqMan PCR, Dis. Aquat. Organ. 60 (2004) 179–187. https://doi.org/10.3354/dao060179.
