# Supplementary material for: Transcriptomic Responses to Koi Herpesvirus in Isolated Blood Leukocytes from Infected Common Carp
Source: Viruses. 2024 Feb 28;16(3):380. doi: 10.3390/v16030380 (PMC10974277; doi:10.3390/v16030380)
Supplement: Supplementary file 1 [file viruses-16-00380-s001.zip › Supplement Table S2.pdf]

Supplement Table S2. The number of Illumina read sequences obtained before and after quality trimming and rRNA removal.

Sample	Number of paired reads	Number of trimmed paired reads	Number of paired reads remaining after rRNA removal	% remaining
C11	33063169	32941606	4523438	13.7
C12	31064617	31010570	4098693	13.2
C9	34718076	34583714	2209940	6.4
K10	32997529	32900575	10829574	32.8
K12	29640142	29556098	10090928	34.0
K9	32216102	32096055	12369925	38.4
